# Decreased ex vivo production of interferon-gamma is associated with severity and poor prognosis in patients with lupus

**DOI:** 10.1186/s13075-017-1404-z

**Published:** 2017-08-25

**Authors:** Sung Soo Ahn, Eun Seong Park, Joo Sung Shim, Sang-Jun Ha, Beom Seok Kim, Seung Min Jung, Sang-Won Lee, Yong-Beom Park, Jason Jungsik Song

**Affiliations:** 1 Division of Rheumatology, Department of Internal Medicine, Yonsei University College of Medicine, 50-1 Yonsei-ro, Seodaemun–gu, Seoul, 03722 South Korea; 2 Department of Biochemistry, College of Life Science & Biotechnology, Yonsei University, Seoul, South Korea; 3 Division of Nephrology, Department of Internal Medicine, Yonsei University College of Medicine, Seoul, South Korea; 4 Institute for Immunology and Immunological Diseases, Yonsei University College of Medicine, Seoul, South Korea

**Keywords:** IFN-γ releasing assay, IFN-γ, Systemic lupus erythematosus, T cell

## Abstract

**Background:**

Lupus pathogenesis is closely associated with interferon gamma (IFN-γ), which plays a central role in innate and adaptive immunity. The aim of this study was to evaluate the ex vivo production of IFN-γ after stimulation of peripheral blood mononuclear cells with phytohemagglutinin (PHA) in patients with lupus, according to disease activity.

**Methods:**

This study included 118 patients with lupus who had undergone IFN-γ-releasing assays (IGRAs) to screen for tuberculosis. Data on IFN-γ production in negative (nil) and positive (mitogen with PHA) controls were collected and analysed. The difference (mitogen minus nil) was used to calculate ex vivo IFN-γ production. Disease activity was evaluated using the Systemic Lupus Erythematosus Disease Activity Index 2000 (SLEDAI-2 K). Poor hospitalisation outcome was defined as in-hospital mortality or intensive care unit admission. Associations among disease activity, poor hospitalisation outcome, and ex vivo IFN-γ production were assessed.

**Results:**

The level of ex vivo IFN-γ production was significantly lower in patients with active systemic lupus erythematosus (SLE) (n = 64) than in those with inactive SLE (n = 54) (median 0.92 vs. 11.06 IU/mL, *p* < 0.001). Ex vivo IFN-γ production was correlated with the SLEDAI-2 K (*r* = − 0.587, *p* < 0.001). Results of multivariate logistic regression analysis showed that ex vivo IFN-γ production ≤ 7.19 IU/mL was an independent predictor for discriminating active and inactive lupus. In addition, patients with ex vivo IFN-γ production ≤ 0.40 IU/mL had more frequent poor hospitalisation outcomes than those with ex vivo IFN-γ production > 0.40 (40.0% vs. 9.3%, *p* = 0.001). The proportion of indeterminate IGRA results was higher in patients with active lupus than in those with inactive lupus (45.3% vs. 0.0%, *p* < 0.001) because of decreased ex vivo IFN-γ production.

**Conclusions:**

Ex vivo IFN-γ production is a useful biomarker for assessing disease activity and predicting poor clinical outcomes of SLE.

**Electronic supplementary material:**

The online version of this article (doi:10.1186/s13075-017-1404-z) contains supplementary material, which is available to authorized users.

## Background

Systemic lupus erythematosus (SLE) is a systemic autoimmune disease that results from a disruption in immune tolerance to self-antigens, leading to inflammation of multiple organs [1]. T cells play a major role in SLE pathogenesis, amplifying inflammation by the secretion of pro-inflammatory cytokines, helping B cells to generate autoantibodies, and maintaining the disease through the accumulation of autoreactive memory T cells [2]. Interferon gamma (IFN-γ) is predominantly produced by T cells and natural killer cells [3], and it plays a critical role in lupus [4]. Previous studies have shown that IFN-γ mRNA expression is increased in peripheral blood mononuclear cells (PBMC) [5], and that serum levels of IFN-γ are elevated in patients with SLE [6, 7]. In addition, in vivo experiments in murine models of SLE have shown that elevations in IFN-γ mRNA levels are correlated with disease progression [8, 9].

Although there is no clinical laboratory test available to examine serum IFN-γ, the IFN-γ-releasing assay (IGRA) is a diagnostic test used to detect *Mycobacterium tuberculosis* (TB) infection by measuring the IFN-γ production by T cells stimulated with TB antigens [10]. Since biologics such as tumour necrosis factor alpha (TNF-α) inhibitors make patients susceptible to reactivation of TB, screening of latent tuberculosis is recommended in the field of rheumatology [11, 12]. IGRA is composed of three tubes (nil, mitogen, and TB antigen) for measuring IFN-γ. The nil tube is a negative control used to measure baseline IFN-γ production, whereas the mitogen tube is a positive control used to measure IFN-γ production with phytohemagglutinin (PHA). Therefore, IGRA results of negative and positive control tubes yield IFN-γ produced at baseline and by the activated T cells, respectively.

IFN-γ production by T cells has not been used to assess disease activity in SLE before; therefore, we evaluated ex vivo IFN-γ production following stimulation with PHA in patients with lupus by analysing the results of IGRA.

## Methods

### Patient selection and study design

We retrospectively analysed the IGRA results of SLE patients who had undergone IGRA at Severance Hospital between November 2009 and December 2016. Patients with a diagnosis of SLE according to the 1997 revised American College of Rheumatology classification criteria [13] were included in the study. The exclusion criteria were as follows: (i) patients with concomitant autoimmune disease; (ii) patients with active infection on the date of the IGRA; (iii) patients with end-stage renal disease; and (iv) patients with absent complement 3 (C3), C4 or anti-double-stranded DNA (dsDNA) results on the date of the IGRA. Ultimately, 118 patients were included in this study. The flowchart for patient selection is shown in Additional file 1: Figure S1. Among the patients with active lupus, 13 patients had follow-up IGRA results available after the lupus became inactive. For comparison, IGRA results from patients with rheumatoid arthritis (RA) were retrospectively obtained for patients who had undergone IGRA before the administration of biologics such as TNF-α inhibitor. Data from healthy controls (*n* = 173) were retrospectively obtained from individuals who had undergone IGRA for a regular health check-up at Severance Hospital. This study was approved by the Institutional Review Board of Severance Hospital (IRB approval number: 4-2016-1115) and conducted in accordance with the principles set forth in the Declaration of Helsinki. The requirement to obtain informed consent was waived because of the retrospective nature of the study.

### Assessment of clinical and laboratory data

The clinical data collected included age, sex, disease duration, clinical manifestations, concurrent immunosuppressive agents, classification of new onset SLE, and the SLE Disease Activity Index-2000 (SLEDAI-2 K) [14]. The disease duration was defined as the period from SLE diagnosis to the date of the initial IGRA, and patients were defined as having new-onset SLE when IGRA was performed within 1 month of the initial diagnosis of SLE. Glucocorticoid dosage was estimated by calculating the total glucocorticoid dosage that was administered 1 week prior to the IGRA, and was expressed in prednisolone equivalent dosage. Clinical manifestations of SLE included skin rash, photosensitivity, oral ulcers, arthritis, serositis, nephritis, and neurological, haematological and immunological disorders, as previously defined [13]. Laboratory data included white blood cell counts; platelets; lymphocyte counts; erythrocyte sedimentation rate (ESR); levels of haemoglobin, C-reactive protein (CRP), blood urea nitrogen (BUN), creatinine (Cr), aspartate aminotransferase (AST), alanine aminotransferase (ALT), total bilirubin, albumin, C3, C4, and anti-dsDNA; and urine protein/creatinine ratio (urine P/Cr).

### Definition of active SLE according to SLEDAI-2 K scores

During the testing period, the SLEDAI-2 K score of each patient was evaluated. Laboratory and clinical abnormalities that were not attributable to SLE were excluded when evaluating SLEDAI-2 K scores. Active SLE was defined as previously described by Franklyn et al. [15]. Patients with SLEDAI-2 K scores ≥ 5 were defined as having active SLE, while patients with SLEDAI-2 K scores < 5 were defined as having inactive SLE. Poor hospitalisation outcome was defined as in-hospital mortality and/or intensive care unit admission.

### Estimation of IFN-γ level assessed by IGRA

For each patient, IGRA was performed in whole blood samples using the QuantiFERON-TB Gold-In Tube test (QFT-GIT; Cellestis, QIAGEN, Germany) according to the manufacturer’s instructions. Briefly, 1 mL of blood was drawn directly into each of the QuantiFERON®-TB Gold blood collection tubes. The kit consists of three blood collection tubes: (i) nil tube (negative control: whole blood without antigens or mitogen); (ii) mitogen tube (positive control: whole blood with phytohemagglutinin); and (iii) TB antigen tube (whole blood with peptides of ESAT-6, CFP-10, and TB7.7 proteins simulating TB-specific antigens). The tubes were incubated overnight at 37 °C, and the concentrations of IFN-γ (IU/mL) were measured using an enzyme-linked immunosorbent assay (ELISA). An automated microplate processor (Evolis Twin Plus system; Bio-Rad Laboratories, Hercules, CA, USA) was used to analyse and calculate the results. Ex vivo IFN-γ production was estimated by calculating the difference in IFN-γ production between the mitogen tube and the nil tube (mitogen minus nil) in order to measure the ability to produce additional IFN-γ after PHA stimulation.

### Statistical analysis

Data analysis was conducted using either GraphPad Prism version 5.0 (GraphPad Software, San Diego, CA, USA) or MedCalc statistical software version 16.2.0 (MedCalc Software bvba, Ostend, Belgium). Data were expressed as medians with inter-quartile ranges (IQR) or for categorical variables, as frequencies and percentages. Continuous variables were compared using Student’s *t* test, and categorical data were compared using the chi-square test or Fisher’s exact test as appropriate. Comparison of nil and ex vivo IFN-γ production in patients with paired IGRA results was performed using the Wilcoxon signed rank test. To compare poor hospitalisation outcome according to ex vivo IFN-γ production, we used Kaplan-Meier analysis and the log-rank test. Correlations between age or disease duration and IFN-γ production in the nil tube, IFN-γ production in the mitogen tube, or ex vivo IFN-γ production, and the correlation between ex vivo IFN-γ production and the SLEDAI-2 K scores were calculated using Pearson’s correlation analysis. Univariate and multivariate logistic regression analyses were performed with forward stepwise logistic regression analysis to compare laboratory variables in differentiating between active and inactive SLE. In multivariate analysis, only variables that were statistically significant in univariate analysis were included. The cut-off value of ex vivo IFN-γ production in discriminating active and inactive SLE and in predicting poor hospitalisation outcome was evaluated using receiver operator characteristic (ROC) curve analysis. In all statistical analyses, a two-tailed *p* value <0.05 was considered statistically significant.

## Results

### Baseline characteristics of patients with SLE with active and inactive disease

Of the 118 patients included in this study, 64 (54.2%) were classified as having active SLE and 54 (45.7%) as having inactive SLE. Patients with active SLE had higher SLEDAI-2 K scores and a larger proportion of them had new-onset SLE. The median age was older in patients with inactive SLE, and the disease duration was longer. The white blood cell count, platelets, lymphocytes, and levels of haemoglobin, albumin, C3, and C4 were lower in patients with active SLE, whereas the ESR, CRP, BUN, Cr, AST, ALT, anti-dsDNA levels, and urine P/Cr ratio were higher (Table 1). The proportion of patients receiving treatment with glucocorticoids, hydroxychloroquine, and azathioprine was larger among patients with inactive SLE, whereas the proportion of patients receiving no immunosuppressive agents was larger among those with active SLE at the time of IGRA testing (Additional file 2: Table S1).

**Table 1:** Baseline characteristics of patients with active and inactive SLE

Variables	Active SLE (*n* = 64)	Inactive SLE (*n* = 54)	*p* value
Demographic data			
Age, years	33.5 (19.0)	41.5 (20.0)	0.029
Female sex, *n* (%)	57 (89.0)	42 (77.7)	0.098
Disease duration, (months)	1.0 (75.5)	62.5 (177.0)	<0.001
SLEDAI-2 K	8.0 (5.0)	2.0 (2.0)	<0.001
New-onset SLE, *n* (%)	33 (51.5)	5 (9.2)	<0.001
Laboratory data			
WBC count (/μL)	3570.0 (3225.0)	4900.0 (3450.0)	0.006
Haemoglobin (g/dL)	9.9 (2.5)	13.2 (2.4)	<0.001
Platelet count (×1000/μL)	158.5 (125.0)	227.0 (111.0)	<0.001
Lymphocyte count (/μL)	580.0 (461.0)	1260.0 (770.0)	<0.001
ESR (mm/h)	46.5 (55.0)	19.5 (23.0)	<0.001
CRP (mg/L)	9.9 (20.3)	0.6 (1.5)	<0.001
BUN (mg/dL)	13.7 (9.9)	12.8 (5.3)	0.025
Cr (mg/dL)	0.6 (0.4)	0.6 (0.2)	0.047
Albumin (mg/dL)	2.9 (1.0)	4.1 (0.5)	<0.001
AST (IU/L)	30.5 (27.5)	19.5 (7.0)	0.028
ALT (IU/L)	18.5 (15.5)	16.5 (13.0)	0.036
Total bilirubin (mg/dL)	0.4 (0.2)	0.5 (0.3)	0.726
Complement 3, mg/dL	50.4 (41.0)	85.0 (33.6)	<0.001
Complement 4, mg/dL	8.5 (9.4)	16.4 (9.3)	<0.001
Anti-dsDNA (IU/mL)	163.5 (360.0)	0.0 (43.0)	<0.001
Urine P/Cr ratio	0.4 (2.8)	0.0 (0.1)	<0.001

Values are expressed as the median (interquartile range) or number (percentage)

*AST* aspartate aminotransferase; *ALT* alanine aminotransferase; *BUN* blood urea nitrogen; *Cr* creatinine, *CRP* C-reactive protein, *ESR* erythrocyte sedimentation rate, *P/Cr* protein/creatinine, SLE systemic lupus erythematosus, *SLEDAI-2 K* Systemic Lupus Erythematosus Disease Activity Index-2000, *WBC* white blood cell

### Comparison of IGRA results in patients with active and inactive SLE

We compared the IGRA results between patients with active and inactive SLE. Interpretation of the results of the IGRA was performed (reported as positive, negative, or indeterminate) and the IFN-γ level was measured in each of the three respective tubes. Although the positivity of the IGRA results was not different between the groups, the proportion of indeterminate results was higher in patients with active SLE than in those with inactive SLE (45.3% vs. 0.0%, *p* < 0.001) (Table 2). Patients with active SLE exhibited higher IFN-γ production in the negative control tube (nil) and the TB antigen tube (median 0.45 vs. 0.09 IU/mL, *p* < 0.001; 0.43 vs. 0.12 IU/mL, *p* = 0.028). However, IFN-γ production in the positive control tube (mitogen) and ex vivo IFN-γ production (mitogen minus nil) was decreased in patients with active SLE (median 2.93 vs. 11.15 IU/mL, *p* < 0.001; 0.92 vs. 11.06 IU/mL, *p* < 0.001) (Table 2). All indeterminate results were due to low ex vivo IFN-γ production. Correlation analysis between age or disease duration and IFN-γ production in the nil tube, IFN-γ production in the mitogen tube, or ex vivo IFN-γ production only revealed negative correlation between age and IFN-γ production in the nil tube (Additional file 3: Table S2). In addition, comparison of immunosuppressive agent usage and indeterminate IGRA results showed that the proportion of patients with indeterminate IGRA results was larger in those who were not receiving any immunosuppressive agents compared to those undergoing concurrent immunosuppressive therapy (19/42 (45.2%) vs. 10/76 (13.1%), *p* < 0.001).

**Table 2:** Comparison of IFN-γ releasing assay (IGRA) results in patients with active and inactive SLE

Variables	Active SLE (*n* = 64)	Inactive SLE (*n* = 54)	*p* value
IGRA results, *n* (%)			
Positive	4 (6.2)	9 (16.6)	0.073
Negative	31 (48.4)	45 (83.3)	<0.001
Indeterminate	29 (45.3)	0 (0.0)	<0.001
IFN-γ level (IU/mL)			
Nil (IU/mL)	0.45 (2.00)	0.09 (0.05)	<0.001
Tuberculosis antigen (IU/mL)	0.43 (1.61)	0.12 (0.21)	0.028
Mitogen (IU/mL)	2.93 (7.09)	11.15 (5.03)	<0.001
Ex vivo IFN-γ production (IU/mL)^a^	0.92 (4.93)	11.06 (5.27)	<0.001

Values are expressed as the median (interquartile range) or number (percentage)

*IFN-γ* interferon gamma, *IGRA* IFN-γ-releasing assay

^a^Ex vivo IFN-γ production was estimated by calculating the difference in IFN-γ production between the mitogen tube and the nil tube (mitogen minus nil)

### Comparison of ex vivo IFN-γ production in patients with SLE, patients with RA, and healthy controls

**Fig. 1:**
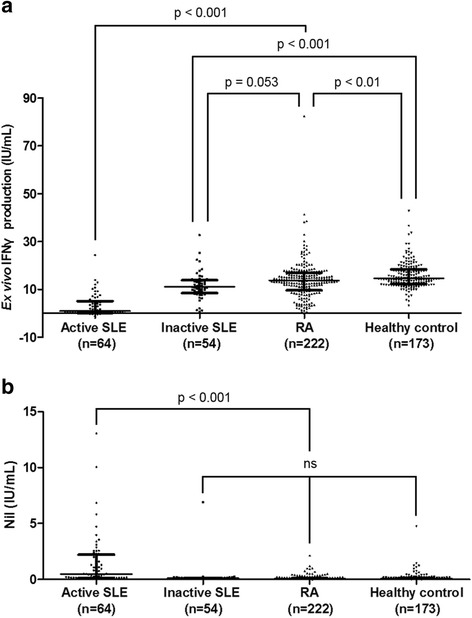
Nil and ex vivo IFN-γ production in patients with SLE or (RA) and healthy controls. Ex vivo IFN-γ production (**a**) and nil (**b**) were compared between patients with active SLE (*n* = 64), inactive SLE (*n* = 54), or RA (*n* = 222), and healthy controls (*n* = 173). Data are expressed as medians, and the error bars indicate interquartile ranges. IFNγ, interferon-gamma, RA rheumatoid arthritis, SLE systemic lupus erythematosus, ns not significant

To evaluate whether decreased ex vivo IFN-γ production is a characteristic finding in SLE, we compared ex vivo IFN-γ production between patients with SLE, patients with RA, and healthy controls. As IGRA testing is routinely recommended before the initiation of biologics, we compared the ex vivo IFN-γ production in patients with SLE with that in patients with active RA who had undergone IGRA testing before initiating biologics. Patients with active SLE had the lowest ex vivo IFN-γ production, followed by those with inactive SLE, those with RA, and then healthy controls. Even though differences in ex vivo IFN-γ production were not observed between patients with inactive SLE and those with RA, patients with RA had lower ex vivo IFN-γ production than healthy controls (RA median 13.78 IU/mL (IQR 9.64–16.99 IU/mL) vs. healthy controls 14.60 IU/mL (IQR 12.34–18.33 IU/mL); *p* < 0.01, Fig. 1a). Patients with active SLE also had increased IFN-γ production in the nil tubes, while differences were not noted between the other groups (Fig. 1b).

### Comparison of ex vivo IFN-γ production before and after immunosuppressive treatment

**Fig. 2:**
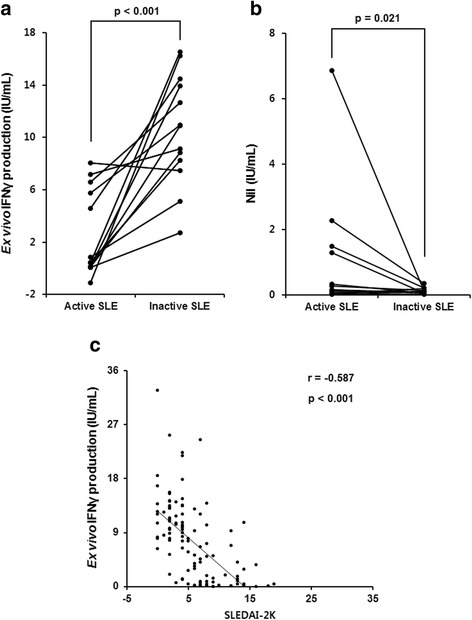
Ex vivo IFN-γ production increases following immunosuppressive treatment in patients with active SLE and correlates with SLEDAI-2 K. Changes in ex vivo IFN-γ production (**a**) and Nil (**b**) after immunosuppressive treatment in 13 patients with follow-up results from the IFN-γ-releasing assay. **c** Correlation between ex vivo IFNγ production and SLEDAI-2 K. IFNγ interferon gamma, SLE systematic lupus erythematosus, SLEDAI-2 K Systemic Lupus Erythematosus Disease Activity Index-2000

We evaluated changes in ex vivo IFN-γ production (mitogen minus nil) and baseline IFN-γ production (nil) in patients with active SLE following treatment. In 13 patients, follow-up IGRA data were available after treatment with immunosuppressive agents, with an interval of at least 6 months. After successful treatment of SLE, ex vivo IFN-γ production increased in 12 out of 13 (92.3%) patients (*p* < 0.001) (Fig. 2a). Similar to the findings shown in Fig. 1b, IFN-γ production in the nil tubes decreased after immunosuppressive treatment (*p* = 0.021) (Fig. 2b). We further evaluated the association between ex vivo IFN-γ production and lupus disease activity measured by the SLEDAI-2 K score. Ex vivo IFN-γ production was negatively correlated with the SLEDAI-2 K score (*r* = − 0.587, *p* < 0.001) (Fig. 2c).

### Clinical utility of ex vivo IFN-γ production in the discrimination of active and inactive SLE

Logistic regression analysis was performed to compare the utility of the laboratory parameters in the differentiation of active and inactive SLE. In the univariate analysis, every laboratory variable except Cr and total bilirubin was shown to be useful in discriminating active and inactive SLE. In addition, using ROC analysis, a cut-off value of ex vivo IFN-γ production ≤ 7.19 IU/mL had an area under the curve of 0.899, with sensitivity of 84.3 and specificity of 87.0 in the discrimination of active and inactive SLE. However, in the multivariate analysis, only ex vivo IFN-γ production ≤ 7.19 (odds ratio (OR) 44.059, 95% confidence interval (CI) 7.315–265.340; *p* < 0.001), albumin (OR 0.087, 95% CI 0.019–0.395; *p* = 0.001), AST (OR 1.196, 95% CI 1.064–1.344; *p* = 0.002), and ALT (OR 0.894, 95% CI 0.829–0.963; *p* = 0.003) were revealed to be useful in discriminating active and inactive SLE (Table 3).

**Table 3:** Comparison of laboratory variables in differentiating active SLE from inactive SLE, using logistic regression analysis

	Univariate analysis			Multivariate analysis		
	Odds ratio	95% CI	*p* value	Odds ratio	95% CI	*p* value
WBC count (/μL)	0.999	0.999–0.999	0.009			
Haemoglobin (g/dL)	0.408	0.298–0.560	<0.001			
Platelet count (×1000/μL)	0.992	0.988–0.997	0.001			
Lymphocyte count (/μL)	0.997	0.996–0.998	<0.001			
ESR (mm/h)	1.032	1.016–1.048	<0.001			
CRP (mg/L)	1.081	1.029–1.137	0.002			
BUN (mg/dL)	1.049	1.002–1.098	0.037			
Cr (mg/dL)	2.964	0.935–9.396	0.064			
Albumin (mg/dL)	0.022	0.005–0.084	<0.001	0.070	0.011–0.441	0.004
AST (IU/L)	1.099	1.048–1.153	<0.001	1.205	1.050–1.383	0.007
ALT (IU/L)	1.023	1.000–1.046	0.047	0.886	0.809–0.971	0.009
Total bilirubin (mg/dL)	1.064	0.747–1.518	0.728			
Complement 3, mg/dL	0.951	0.934–0.969	<0.001			
Complement 4, mg/dL	0.910	0.868–0.955	<0.001			
Anti-dsDNA (IU/mL)	1.009	1.005–1.014	<0.001			
Urine P/Cr ratio	5.874	1.772–19.470	0.003			
Ex vivo IFN-γ production (IU/mL)	0.721	0.646–0.804	<0.001			
Ex vivo IFN-γ production ≤ 7.19 IU/mL	36.257	12.789–102.788	<0.001	49.004	5.871–408.991	<0.001
Concurrent immunosuppressive treatment	0.054	0.017–0.170	<0.001	0.056	0.006–0.469	0.007

*AST* aspartate aminotransferase, *ALT* alanine aminotransferase, *BUN* blood urea nitrogen, *Cr* creatinine, *CRP* C-reactive protein, *ESR* erythrocyte sedimentation rate, *IFN-γ* interferon gamma, *P/Cr* protein/creatinine, *SLE* systemic lupus erythematosus, *WBC* white blood cell

### Comparison of poor hospitalisation outcome according to ex vivo IFN-γ production

**Fig. 3:**
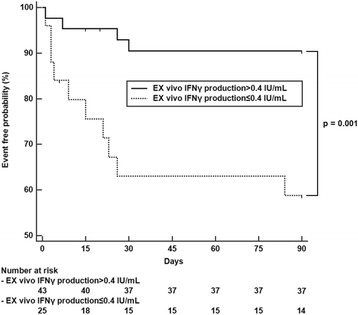
Comparison of poor hospitalisation outcomes according to ex vivo IFN-γ production. The proportion of patients with poor hospitalisation outcomes was larger among patients with ex vivo IFN-γ production ≤ 0.40 IU/mL than in those with ex vivo IFN-γ production > 0.40 IU/mL. Poor hospitalisation outcome was defined as in-hospital mortality and/or intensive care unit admission. IFN-γ interferon gamma

Among the patients included in this study, 68 had undergone IGRA testing during the admission period. Fourteen patients had poor hospitalisation outcome. Six patients had in-hospital mortality, whereas eight were admitted to the intensive care unit. Results of the ROC curve analysis showed that ex vivo IFN-γ production ≤ 0.40 IU/mL was the best cut-off for predicting poor hospitalisation outcome. Results of Kaplan-Meier analysis with the log-rank test showed that patients with ex vivo IFN-γ production ≤ 0.40 IU/mL had more frequent poor hospitalisation outcomes than those with ex vivo IFN-γ production > 0.40 IU/mL (10/25 (40.0%) vs. 4/43 (9.3%); *p* = 0.001) (Fig. 3).

## Discussion

Although IFN-γ mainly mediates host defence against microbial invasion, it is known to have a pivotal role in SLE. The inhibition of IFN-γ has shown benefits in reducing disease activity in murine models of SLE [16, 17] and a therapeutic monoclonal antibody against IFN-γ is being developed for the treatment of SLE [18, 19]. In this study, we evaluated IFN-γ production in patients with SLE, using IGRA results obtained during the screening of latent TB prior to immunosuppressive treatment. Our data demonstrated that ex vivo production of IFN-γ is decreased in patients with active disease compared to patients with inactive disease. The results of our study are different from those of previous publications that have reported increased IFN-γ production or IFN-γ-related gene expression in SLE, because our study evaluated IFN-γ production after PHA stimulation in whole blood [6, 7, 20].

However, our data are not contradictory to previous findings, in that baseline IFN-γ production (nil results) in patients with active SLE is increased compared to that in patients with inactive SLE, in patients with RA, and in healthy controls. While baseline IFN-γ production increased, ex vivo IFN-γ production after stimulation with PHA decreased in patients with active SLE. In a previous study by Hagiwara et al., ex vivo experiments using enzyme-linked immunospot (ELISPOT) assay demonstrated that IFN-γ-producing T cells were decreased in patients with active lupus [21], which is similar to our finding. Our study used ELISA, which can measure the total amount of ex vivo IFN-γ production in whole blood, excluding baseline production. Since ELISA is known for its high sensitivity and wide dynamic range, measuring ex vivo IFN-γ production by ELISA can be a suitable biomarker for disease activity in lupus. Decreased ex vivo IFN-γ production could be associated with T cell exhaustion, which is a non-functional state that occurs under conditions of antigen persistence, such as those that arise during various infections and cancers [22, 23]. Similar to infection and cancer, T cell exhaustion has also been described in SLE [24]. Excessive auto-antigen exposure in active SLE may lead to T cell exhaustion. Alternatively, T cells may become unresponsive due to a negative feedback mechanism during periods of overwhelming inflammation. PD-1, an inhibitory T cell marker, is increased in SLE [25]. Likewise, critically ill patients who are admitted to the intensive care unit have been shown to demonstrate a high proportion of indeterminate results on the IGRA due to unresponsiveness to mitogen stimulation [26].

Our observations have raised an important issue regarding the limitations of the IGRA in SLE. Nearly half of all patients with active SLE had indeterminate results from the IGRA, while none of the patients with inactive SLE had an indeterminate IGRA result. The IGRA results are dependent on IFN-γ production by TB antigen-specific T cells. However, when IFN-γ production is hampered by defective T cells, the sensitivity of the IGRA may be reduced. Unreliable IGRA results in patients with T cell defects have been reported in HIV infection [27]. Our study suggests that the interpretation of the IGRA requires caution in patients with active SLE.

More importantly, decreased ex vivo IFN-γ production correlates well with SLEDAI-2 K scores, and the poor hospitalisation outcome was more frequent in patients with ex vivo IFN-γ production ≤ 0.40 IU/mL. The findings of our study imply that monitoring ex vivo IFN-γ production could aid in assessing disease activity and predicting clinical outcome in SLE. So far, no test is available to evaluate T cell reactivity for SLE disease activity and prognosis, and our data provide the possibility of developing a diagnostic test to measure ex vivo IFN-γ production. Furthermore, we demonstrated that decreased production of IFN-γ in patients with active SLE recovers when SLE becomes inactive following immunosuppressive treatment. Similar to our findings for SLE, decreased IFN-γ production in patients with RA has been shown to recover after TNF-α inhibitor treatment [28]. Decreased ex vivo IFN-γ production was likewise noted in our group of patients with active RA as compared with healthy controls. However, decreased ex vivo IFN-γ production was more pronounced in active SLE than in active RA in our study.

The strength of our study is that we included a large number of subjects who had undergone IGRA, an ex vivo method to estimate IFN-γ production in patients with SLE. However, the present study has several limitations. First, the clinical and laboratory data and IGRA results of patients were collected by reviewing the medical records. Since the IGRA is not routinely performed in patients with SLE, there could have been patient selection bias, resulting in the selection of patients with more severe SLE in our study. Therefore, our study findings should be validated in future prospective studies. Second, the effect of immunosuppressive treatment was not thoroughly controlled. It is possible that immunosuppressive treatment affects the decrease in ex vivo IFN-γ production. However, the proportion of patients with active SLE treated with glucocorticoids or immunosuppressive agents was lower than that of patients with inactive SLE, as most patients with SLE had undergone IGRA before initiating potent immunosuppressive treatment. Therefore, the effect of immunosuppressive treatment on active lupus may not be significant. Third, we did not evaluate CD4 T cell numbers. Although we demonstrated that ex vivo IFN-γ production ≤ 7.19 IU/mL was an independent predictor for discriminating active and inactive lupus regardless of leukopenia and lymphopenia, there is a possibility that the level of ex vivo IFN-γ production is associated with CD4 T cell numbers.

## Conclusions

In conclusion, we have demonstrated that ex vivo IFN-γ production decreases in active SLE, which correlates with SLEDAI-2 K scores. In addition, the prognosis of patients with SLE with low ex vivo IFN-γ production was unfavourable. These findings suggest that *e*x vivo IFN-γ production might be a useful biomarker for monitoring disease activity in patients with SLE. Furthermore, special caution in the interpretation of results is required when IGRA is performed in patients with active lupus because of a large proportion of indeterminate results.

## Additional files

Additional file 1: Figure S1. Flowchart for patient selection in the present study. (TIF 120 kb)

Additional file 2: Table S1. Comparison of clinical manifestations and immunosuppressive agents in patients with active and inactive SLE. (DOCX 15 kb)

Additional file 3: Table S2. Correlation analysis of age and disease duration with IFN-γ production in the nil tube, mitogen tube, and *ex vivo* IFN-γ production. (DOCX 14 kb)

## Abbreviations

ALT: Alanine aminotransferase
AST: Aspartate aminotransferase
BUN: Blood urea nitrogen
C3: Complement component 3
C4: Complement component 4
CI: Confidence interval
Cr: Creatinine
CRP: C-reactive protein
ELISA: Enzyme-linked immunosorbent assay
ESR: Erythrocyte sedimentation rate
IFN-γ: Interferon gamma
IGRA: Interferon-gamma-releasing assays
NK: Natural killer
OR: Odds ratio
P/Cr: Protein/creatinine
PBMC: Peripheral blood mononuclear cells
PHA: Phytohemagglutinin
RA: Rheumatoid arthritis
ROC: Receiver operator characteristic
SLE: Systemic lupus erythematosus
SLEDAI-2 K: Systemic lupus erythematosus disease activity index-2000
TB: *Mycobacterium tuberculosis*
TNF: Tumour necrosis factor
WBC: White blood cell
