# Supplementary figures and images for: Decreased ex vivo production of interferon-gamma is associated with severity and poor prognosis in patients with lupus
Source: Arthritis Res Ther. 2017 Aug 25;19:193. doi: 10.1186/s13075-017-1404-z (PMC5574096; doi:10.1186/s13075-017-1404-z)

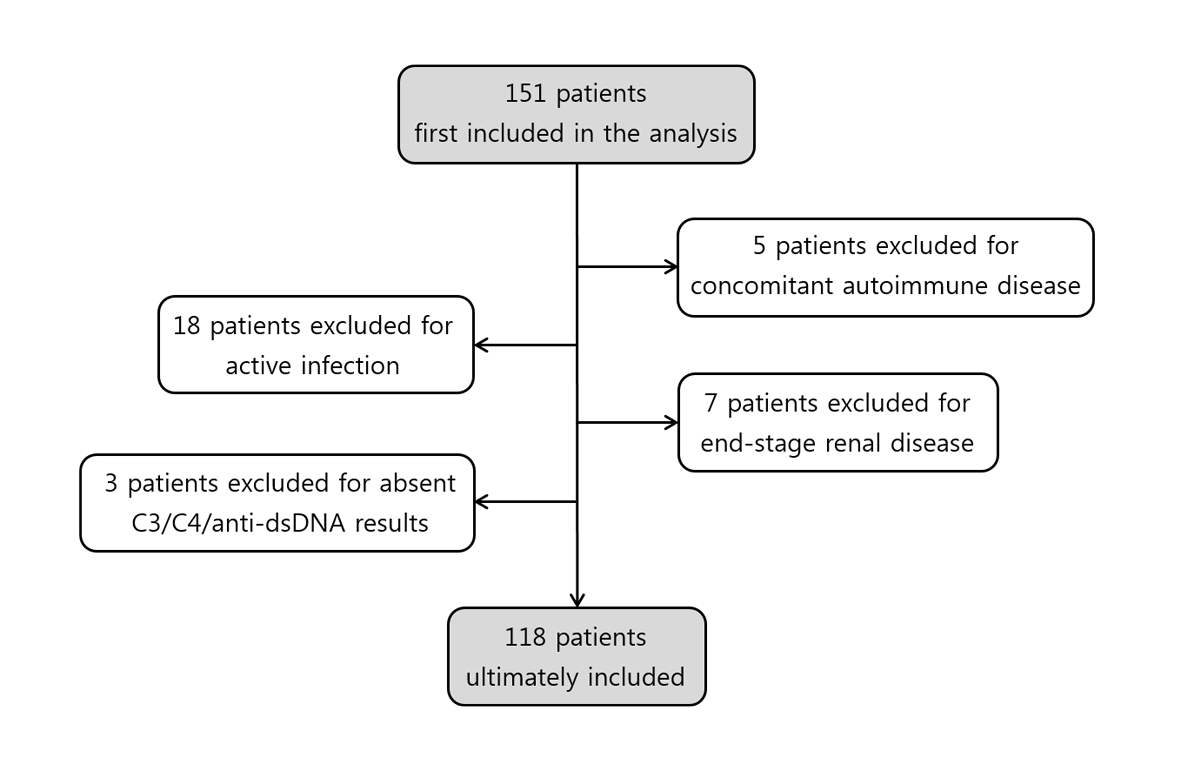

Supplement: Supplementary file 1 — Flowchart for patient selection in the present study. (TIF 120 kb) [file 13075_2017_1404_MOESM1_ESM.tif]
