# Supplementary material for: Decreased ex vivo production of interferon-gamma is associated with severity and poor prognosis in patients with lupus
Source: Arthritis Res Ther. 2017 Aug 25;19:193. doi: 10.1186/s13075-017-1404-z (PMC5574096; doi:10.1186/s13075-017-1404-z)
Supplement: Supplementary file 2 — Comparison of clinical manifestations and immunosuppressive agents in patients with active and inactive SLE. (DOCX 15 kb) [file 13075_2017_1404_MOESM2_ESM.docx]

**Additional file 2: Table S1. Comparison of clinical manifestations and immunosuppressive agents in patients with active and inactive SLE**

|  | Active SLE (n=64) | Inactive SLE (n=54) | p-value |
| --- | --- | --- | --- |
| Clinical manifestations, n (%)^†^ |  |  |  |
| Skin rash | 19 (29.6) | 1 (1.8) | <0.001 |
| Photosensitivity | 0 (0.0) | 0 (0.0) | 0.999 |
| Oral ulcers | 8 (12.5) | 0 (0.0) | 0.007 |
| Arthritis | 4 (6.2) | 0 (0.0) | 0.124 |
| Serositis | 30 (46.8) | 1 (1.8) | <0.001 |
| Nephritis | 31 (48.4) | 4 (7.4) | <0.001 |
| Neurological disorder | 5 (7.8) | 0 (0.0) | 0.061 |
| Haematological disorder | 63 (98.4) | 37 (68.5) | <0.001 |
| Immunological disorder | 60 (93.7) | 26 (48.1) | <0.001 |
| Immunosuppressive  agents |  |  |  |
| Glucocorticoid | 26 (40.6) | 42 (77.7) | <0.001 |
| Glucocorticoid dosage^a^ (mg) | 21.8 (70.0) | 35.0 (70.0) | 0.698 |
| Hydroxychloroquine | 13 (20.3) | 35 (64.8) | <0.001 |
| Mycophenolate mofetil | 5 (7.8) | 10 (18.5) | 0.083 |
| Azathioprine | 1 (1.5) | 6 (11.1) | 0.046 |
| Tacrolimus/cyclosporine | 2 (3.1) | 5 (9.2) | 0.244 |
| Methotrexate | 0 (0.0) | 1 (1.8) | 0.457 |
| Cyclophosphamide | 0 (0.0) | 0 (0.0) | 0.999 |
| None | 38 (59.3) | 4 (7.4) | <0.001 |

Values are expressed as the median (interquartile range) or n (%).

^†^The presence of clinical manifestations was evaluated according to the 1997 revised American College of Rheumatology classification criteria.

^a^Glucocorticoid dosage was estimated by calculating the total glucocorticoid dosage that was administered 1 week prior to the IGRA.
