# Supplementary material for: Decreased ex vivo production of interferon-gamma is associated with severity and poor prognosis in patients with lupus
Source: Arthritis Res Ther. 2017 Aug 25;19:193. doi: 10.1186/s13075-017-1404-z (PMC5574096; doi:10.1186/s13075-017-1404-z)
Supplement: Supplementary file 3 — Correlation analysis of age and disease duration with IFN-γ production in the nil tube, mitogen tube, and ex vivo IFN-γ production. (DOCX 14 kb) [file 13075_2017_1404_MOESM3_ESM.docx]

**Additional file 3: Table S2. Correlation analysis of age and disease duration with IFNγ production in the nil tube and mitogen tube, and *ex vivo* IFNγ production**

|  | Age | Disease duration |
| --- | --- | --- |
| Nil tube (IU/mL) | -0.243 (0.008) | -0.057 (0.534) |
| Mitogen tube (IU/mL) | 0.082 (0.377) | 0.126 (0.173) |
| *Ex vivo* IFNγ production (IU/mL) | 0.152 (0.100) | 0.140 (0.128) |

Data in tables are expressed as a correlation coefficient with p-values in parenthesis.
